# Reduced cingulate gyrus volume in Cavalier King Charles Spaniels with syringomyelia and neuropathic pain revealed by voxel-based morphometry: a pilot study

**DOI:** 10.3389/fnana.2023.1175953

**Published:** 2023-07-17

**Authors:** Björn Nitzsche, Sabine Schulze, Johannes Boltze, Martin J. Schmidt

**Affiliations:** ^1^Department of Nuclear Medicine, University Hospital Leipzig, Leipzig, Germany; ^2^Faculty of Veterinary Medicine, Institute of Anatomy, Histology and Embryology, University of Leipzig, Leipzig, Germany; ^3^Department of Veterinary Clinical Sciences, Small Animal Clinic, Neurosurgery, Neuroradiology and Clinical Neurology, Justus-Liebig-University, Giessen, Germany; ^4^Small Animal Clinic, Department of Veterinary Medicine, Free University of Berlin, Berlin, Germany; ^5^School of Life Sciences, University of Warwick, Coventry, United Kingdom

**Keywords:** hyperesthesia, brain, syringomyelia, Chiari malformation, dog

## Abstract

**Objective:**

Pathomorphological alterations of the central nervous system in dogs, such as syringomyelia and Chiari-like malformation, can cause cranial and cervical hyperesthesia and neuropathic pain. The long-term activity of the pain network can induce functional alteration and eventually even morphological changes in the pain network. This may happen especially in the prefrontal and cingulate cortex, where atrophy of the gray matter (GM) was observed in humans with chronic pain, irrespective of the nature of the pain syndrome. We tested the hypothesis that Cavalier King Charles Spaniels (CKCS) with Chiari-like malformation and associated syringomyelia (SM) and pain show cerebral morphological differences compared to animals without signs of syringomyelia and pain.

**Methods:**

Volumetric datasets of 28 different brain structures were analyzed in a retrospective manner, including voxel-based morphometry, using magnetic resonance imaging data obtained from 41 dogs.

**Results:**

Volumetric analyses revealed a decrease in GM volumes in the cingulate gyrus (CG) in CKCS with SM and chronic pain when normalized to brain volume. This finding was supported by voxel-based morphometry, which showed a cluster of significance within the CG.

**Conclusion:**

GM atrophy in the CG is associated with chronic pain and thus may serve as an objective readout parameter for the diagnosis or treatment of canine pain syndromes.

## Introduction

**Figure 1:**
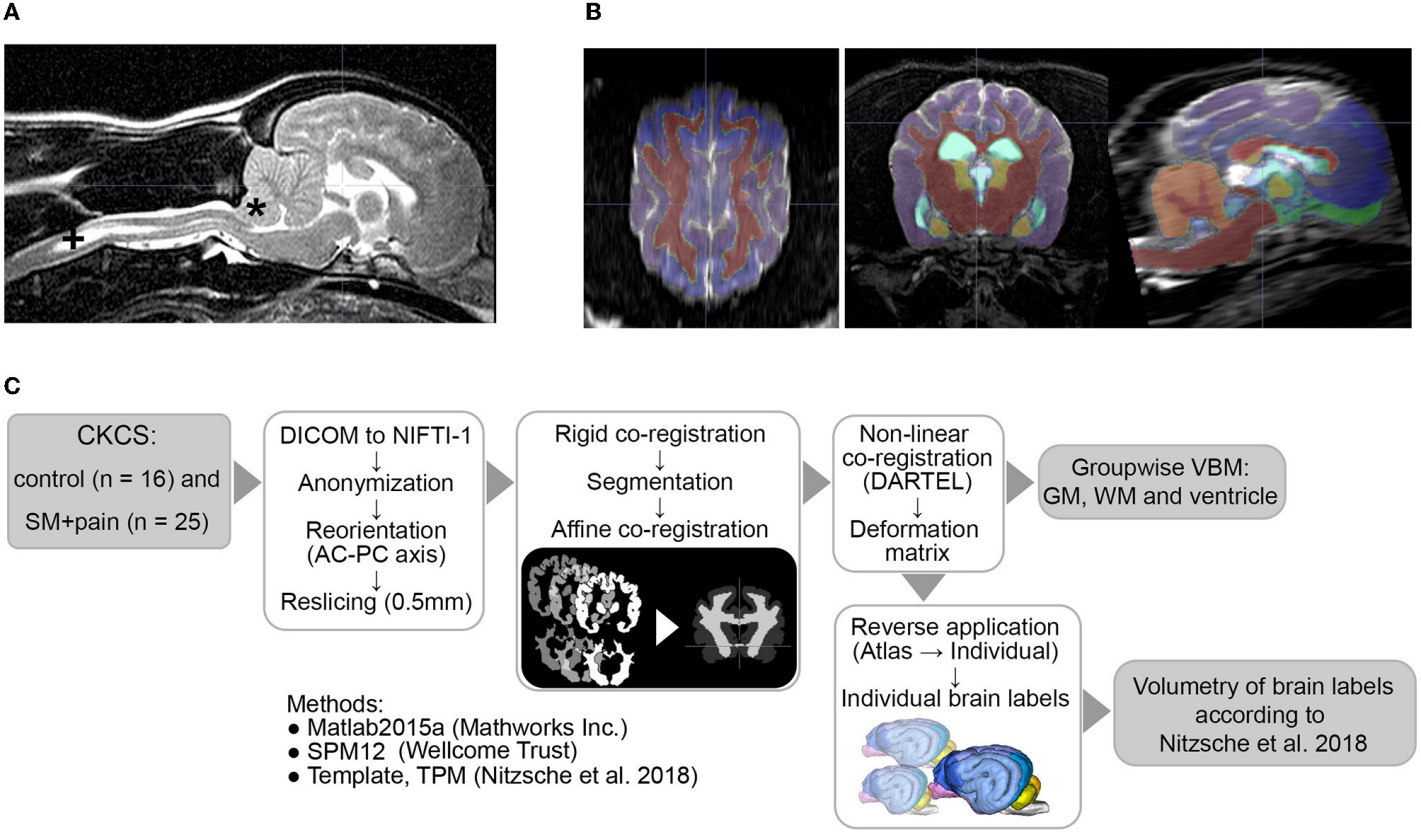
**(A)** MRI findings of an individual Chiari-like malformation Type I (*) in a Cavalier King Charles Spaniel (CKCS) with syringomyelia (+, SM) and pain, **(B)** atlas labels aligned to individual MR data, and **(C)** image postprocessing and analyses. AC-PC, anterior commissure/posterior commissure; TPM, tissue probability maps; DARTEL, Diffeomorphic Anatomical Registration Through Exponentiated Lie Algebra; VBM, voxel-based morphometry; GM, gray matter; WM, white matter.

Syringomyelia (SM) is a debilitating pathological condition of the spinal cord that is frequently diagnosed in Cavalier King Charles Spaniels (CKCS). SM is often associated with a Chiari-like malformation (CM; Rusbridge et al., 2000; Loderstedt et al., 2011). This canine variant of the human Chiari malformation type 1 includes a shortened basioccipital bone, basal invagination, and atlantooccipital overlap, as well as atlantoaxial instability (Rusbridge et al., 2000). A premature closure of the basioccipital synchondrosis in the skull base was suggested to cause basicranial shortening and morphological alterations of the entire skull (Schmidt et al., 2013a,b, 2014). As a result, the longitudinal axis of the brain gets compressed, and the caudal aspect of the cerebellum deviates into the foramen magnum (Figure 1A). This results in jet streams of cerebrospinal fluid (CSF) at the craniocervical junction directed into the subarachnoid space and central canal and causes segmental spinal cord expansion, tissue shear, and parenchymal damage, ultimately leading to pathognomonic CSF-filled cavities (Hu et al., 2012; Cirovic et al., 2018). Dogs with CM can have a normal spinal cord at the beginning of their lives and develop SM over several years. For unknown reasons, SM is not associated with clinical signs in some dogs, while SM creates neuropathic pain expressed as cranial and cervical hyperesthesia (on palpation or spontaneously) and spontaneous scratching associated with sometimes excessive vocalization and touch aversion in other dogs (Rusbridge et al., 2019). The neuropathological correlates of neuropathic pain in dogs include affection of the dorsal horn cells of the spinal cord gray matter (GM) that carries sensory information from the body to the brain (Rusbridge and Jeffery, 2008; Schmidt et al., 2013a,b), as well as intraparenchymal inflammatory processes (Hu et al., 2012; Schmidt et al., 2013a,b).

In contrast to other forms of sensory input, there is no specific cortical area dedicated to nociceptive sensory input processing (Ahmad and Abdul Aziz, 2014). Pain experience rather results from coordinated activity in several brain regions, referred to as the pain network (Garcia-Larrea and Peyron, 2013). While the somatosensory cortex processes the primary discriminatory components of pain perception such as modality and location (Flor et al., 1995; Bushnell et al., 2013), other brain structures in this network (insular cortex, thalamus, medial- and orbitofrontal areas of the prefrontal cortex, cingulate cortex, and periaqueductal GM) are responsible for vegetative, emotional, and cognitive responses to pain (Price, 2000; Apkarian et al., 2004; Bushnell et al., 2013). The pain network also facilitates an effective modulation of pain perception in both humans with chronic pain syndromes (May, 2008) and animals following experimental chronic pain induction (Stephen McMahon et al., 2013). The prefrontal cortex, as an element of the pain network, can activate the periaqueductal GM, raphe nuclei, and other medullary centers, which in turn send descending inhibitory signals to the dorsal horn of the spinal cord for modulation of pain transmission (Moore, 2016). Activation of the prefrontal cortex is suggested to be responsible for the psycho-analgesic effects of placebos and hypnosis that are capable of reducing the intensity of perceived pain (Moriarty et al., 2011). However, long-term activity can induce functional alteration and eventually even morphological changes in the pain network, especially in the prefrontal and cingulate cortices (Zhuo, 2008; Rodriguez-Raecke et al., 2009). Atrophy of the GM was observed in various parts of the pain network in humans with chronic pain, irrespective of the nature of the pain syndrome (May, 2008; Li et al., 2010; Obermann et al., 2013; Smallwood et al., 2013; Cauda et al., 2014; Fritz et al., 2016). The observed cortical GM decline is suggested to impair effective antinociception and, possibly, to increase central sensitization, thereby decreasing the pain threshold (Zhuo, 2008). Since parts of the pain network also play important roles in general emotional, motivational, and cognitive processes (Heitmann et al., 2022), cortical GM atrophy can also result in comorbidities of chronic pain such as anxiety and depression, impaired working memory, and executive dysfunction in both humans (Moriarty et al., 2011) and animals (Hu et al., 2010).

The association between chronic pain and cortical GM atrophy in the pain network was shown in laboratory rodents with chronic pain (Sang et al., 2018; Zhou et al., 2022). However, there is no data on animals with naturally occurring chronic pain syndromes or non-laboratory animals. In this study, we investigate possible differences in regional brain volumes in CKCS with a Chiari-like malformation and associated SM/chronic neuropathic pain compared to healthy control CKCS.

## Materials and methods

### Animals

The medical records of all CKCS with CM that underwent magnetic resonance imaging (MRI) of the head and cervical spine in the Clinic for Small Animals at the Justus Liebig University of Giessen, Germany, between 2011 and 2016 were reviewed retrospectively. MRI was either performed for breeding selection against SM at the request of the animals' owners, or the dogs were examined for diagnostic workup of clinical signs such as hyperesthesia or pain. The medical history and results of general physical and neurological examinations were also reviewed. MRI datasets from two groups were examined. First, the control group included clinically healthy CKCS without SM and without any documented clinical signs of acute or chronic pain (*n* = 16). Second, the study group was composed of dogs with SM exhibiting clinical symptoms of neuropathic pain for at least 3 months (*n* = 25).

### Ethical statement

MRI data were originally obtained for diagnostic purposes and analyzed retrospectively. Therefore, approval from the ethics committee of the Justus Liebig University and the responsible regional governmental authority (Regierungspräsidium) of the federal state of Hesse was not sought because the authority waives ethical review of retrospective studies based on clinical imaging conducted for diagnostic purposes.

### Imaging technique

A standard anesthetic protocol was used (isoflurane inhalation narcotic 1.5%−2.5%, premedication with diazepam 0.1 mg/kg, and propofol 6 mg/kg). Image acquisition was performed using a 1.0 Tesla Scanner (Gyroscan Intera, Phillips, Hamburg, Germany) with a SENSE-flex-M coil placed bilaterally. Sagittal, transverse, and dorsal scans of the entire brain and the cervical spine were performed. T2-weighted (T2-Turbospin echo, TE/TR: 108/8,627 ms, averages: 4; slice thickness: 2.0 mm, spacing: 2.2 mm, acquisition matrix: 288 × 288; flip angle: 90°; voxel size: 0.4 × 0.4 × 2.2 mm, acquisition time: 16 min) sections of the brain were used for further investigations.

### Image processing and analysis

Image processing was performed as described previously (Nitzsche et al., 2018) by a researcher blinded to the groups. The workflow is given in Figure 1. Briefly, all DICOM datasets were anonymized and stored in NIFTI-1 format. The data were reoriented according to canine stereotaxic space (Nitzsche et al., 2018) and realigned/resliced to 0.5 mm isometric voxel size according to canine atlas space using 6th-order B-spline interpolation. Thereafter, the data sets were rigidly co-registered (6th degree of freedom) to the canine T2w TSE breed-averaged template using SPM12 (Version 7771, Welcome Trust Center) in Matlab 2019 (Mathworks Inc.). All data were then automatically segmented into GM, white matter (WM), and CSF by the SPM12 routine, and canine tissue probability maps were created as described previously using a Markov Random Field cleanup (Nitzsche et al., 2018, Markov Random Field cleanup: 2). Individual GM, WM, and ventricular tissue masks were affinely co-registered (normalized, sampling distance: 2 mm) and, subsequently, non-linearly co-registered to the canine brain template mask using the Diffeomorphic Registration Algorithm (DARTEL, Ashburner, 2007).

### Volumetry

The calculated individual deformation matrices were reversely applied to the modified canine brain atlas labels (Nitzsche et al., 2018) to transform the labels into an individual space. The atlas labels included GM of the cerebral hemispheres (the frontal, temporal, parietal, and occipital lobe), olfactory bulb, cingulate gyrus (CG), subcortical GM (the caudate nucleus, thalamus, and lateral and medial geniculate body), and hippocampus, as well as WM tracts (the lateral olfactory tract and corpus callosum), cerebellar structures (the lateral hemispheres, vermis, and cerebellar white matter), and internal CSF spaces (the lateral ventricle, 3rd ventricle, mesencephalic aqueduct, 4th ventricle, and central canal). The volume was calculated in mL and in the percentage of the respective brain volume from each brain label. The quality of reverse atlas deformation and segmentation was visually verified by the responsible researcher (Figure 1B).

### Voxel-based morphometry

To correct for local spatial differences, Diffeomorphic Anatomical Registration Through Exponentiated Lie Algebra (DARTEL)-registered GM and ventricle masks were initially smoothed using a 3 mm Gaussian filter and subsequently analyzed using the SPM12 pipeline. Voxel-based morphometry (VBM) for GM and ventricles was performed using a *t*-test with family-wise correction with brain volume, age, and bodyweight as covariates (Whitwell, 2009; Abbott et al., 2012).

### Statistical analysis

Statistical analysis of absolute and relative volumes was performed using a commercial software package (Graph Pad Prism 4.0, Graph Pad Software Inc., San Diego, California). The deviation from the normal distribution was checked using the normal probability plot of the model residuals for each variable. A one-way Analysis of Covariance (ANCOVA) with respect to bodyweight and multicomparison using the Bonferroni method was performed. The *p*-values of ≥0.05 and <0.1 were interpreted as indicating trends. A family-wise significance level was used for all comparisons. The *p*-value of <0.05 was considered statistically significant. The data are presented as mean ± standard deviation. The Quartile Coefficient of Dispersion (QCD) was calculated for all relative label volumes using the formula: QCD = (Quartile 3 – Quartile 1)/median.

## Results

Table 1 provides summarized data on the study population, including sex, body weight, and age. A table with detailed population data is included in the supportive data section (Supplementary Table 1). There were no statistically significant differences between both groups regarding age (*p* = 0.117) or body weight (*p* = 0.278). Clinical signs in the 25 CKCS with SM and chronic neuropathic pain included several paroxysmal pain manifestations with vocalization and owner-reported phantom scratching directed at the cervical or shoulder area (mean frequency: 5/day, range from 2/week to 20/day). The duration of clinical signs ranged from 3.5 to 24 months (mean 8.3 months). There was a moderate correlation between the duration of clinical signs and the frequency of pain attacks (*r*^2^ = 0.57; *p* < 0.05).

**Table 1:** Demographic data of study groups.

	**CKCS with SM and pain**	**Control**
Sex (female/male)	9/16	10/6
Weight in kg	9.3 ± 1.5	8.8 ± 1.2
Age in months	57.3 ± 24.5	45.2 ± 19.1

CKCS, Cavalier King Charles Spaniels; SM, syringomyelia.

### Absolute brain volume comparison

No significant differences could be observed for temporal, parietal, occipital, frontal, and olfactory lobe volumes between controls and CKCS with SM and chronic pain. Also, there were neither statistically significant differences in the absolute volumes of the lateral, 3rd, and 4th ventricles nor in the mesencephalic aqueduct volumes. Volumes of the caudate nucleus, thalamus, hippocampus, and lateral geniculate body, as well as cortical and cerebellar volumes, were also indifferent. However, controls exhibited significantly smaller corpus callosum and lateral olfactory tract volumes compared to CKCS with SM and pain. Moreover, the absolute WM volume in the control group was significantly smaller than in CKCS with SM and pain. Please refer to Table 2 for details.

**Table 2:** Comparison of absolute tissue volume in ml and relative tissue volumes in the percentage of individual total brain volume in CKCS.

**Tissue volume**	**CKCS with SM and pain**	**Control**	***p*-value absolute/relative**
Total brain	56.2 ± 4.9 ml	52.6 ± 3.8 ml	0.122
White matter	16.8 ± 1.4 ml 30.0 ± 2.3%	15.5 ± 1.1 ml 29.5 ± 1.7%	0.011^*^/0.804
Gray matter	30.9 ± 3.4 ml 54.8 ± 2.1%	29.2 ± 2.5 ml 55.6 ± 2.1%	0.556/0.621
Total ventricle	5.1 ± 0.8 ml 9.1 ± 1.6%	5.2 ± 0.9 ml 9.9 ± 1.6%	0.869/0.294
Cerebellum	5.3 ± 0.5 ml 9.4 ± 0.3%	4.9 ± 0.4 ml 9.2 ± 0.5%	0.086^#^/0.339
Temporal lobe	11.8 ± 1.1 ml (21.0 ± 0.8%)	11.1 ± 0.9 ml (21.1 ± 0.7%)	0.118/0.705
Parietal lobe	4.4 ± 0.5 ml (7.9 ± 0.4%)	4.3 ± 0.4 ml (8.2 ± 0.5%)	0.750/0.088^#^
Cingulate gyrus	2.0 ± 0.2 ml (3.5 ± 0.1%)	1.9 ± 0.2 ml (3.7 ± 0.2%)	0.698/0.007^**^
Occipital lobe	4.5 ± 0.6 ml (7.9 ± 0.5%)	4.2 ± 0.4 ml (8.0 ± 0.4%)	0.234/0.780
Frontal lobe	5.8 ± 0.8 ml (10.3 ± 0.7%)	5.5 ± 0.6 ml (10.4 ± 0.8%)	0.324/0.853
Olfactory lobe	1.9 ± 0.3 ml (3.4 ± 0.3%)	1.7 ± 0.2 ml (3.3 ± 0.2%)	0.062^#^/0.309
Caudate nucleus	0.7 ± 0.2 ml (1.3 ± 0.2%)	0.7 ± 0.1 ml (1.3 ± 0.1%)	0.180/0.527
Thalamus	0.7 ± 0.1 ml (1.2 ± 0.2%)	0.6 ± 0.1 ml (1.2 ± 0.1%)	0.168/0.522
Hippocampus	1.2 ± 0.2 ml (2.1 ± 0.2%)	1.1 ± 0.2 ml (2.1 ± 0.4%)	0.434/0.590
Cerebellar hemisphere	2.4 ± 0.3 ml (4.2 ± 0.2%)	2.2 ± 0.3 ml (4.2 ± 0.5%)	0.200/0.927
Vermis	1.8 ± 0.2 ml (3.2 ± 0.2%)	1.6 ± 0.2 ml (3.1 ± 0.2%)	0.133^#^/0.729
Cerebellar white matter	1.1 ± 0.1 ml (2.0 ± 0.2%)	1.0 ± 0.2 ml (1.9 ± 0.4%)	0.162/0.799
Lateral geniculate body	0.2 ± 0.1 ml (0.4 ± 0.01%)	0.2 ± 0.02 ml (0.4 ± 0.01%)	0.068^#^/0.325
Corpus callosum	0.6 ± 0.1 ml (1.1 ± 0.1%)	0.6 ± 0.1 ml (1.1 ± 0.1%)	0.009^**^/0.512
Olfactory tract	0.34 ± 0.04 ml (0.61 ± 0.1%)	0.30 ± 0.03 ml (0.57 ± 0.1%)	0.003^**^/0.356
Lateral cerebral ventricle	3.7 ± 0.1 ml (6.6 ± 1.3%)	3.8 ± 0.8 ml (7.2 ± 1.4%)	0.772/0.300
3rd ventricle	0.6 ± 0.1 ml (1.1 ± 0.2%)	0.6 ± 0.1 ml (1.2 ± 0.1%)	0.854/0.344^#^
4th ventricle	0.3 ± 0.02 ml (0.5 ± 0.04%)	0.3 ± 0.02 ml (0.5 ± 0.04%)	0.524/0.140
Mesencephalic aqueduct	0.3 ± 0.03 ml (0.5 ± 0.1%)	0.3 ± 0.03 ml (0.6 ± 0.1%)	0.965/0.105

CKCS, Cavalier King Charles Spaniels; SM, syringomyelia.

# p ≤ 0.1.

* p < 0.05.

** p < 0.01.

### Relative structure volumes and VBM

**Figure 2:**
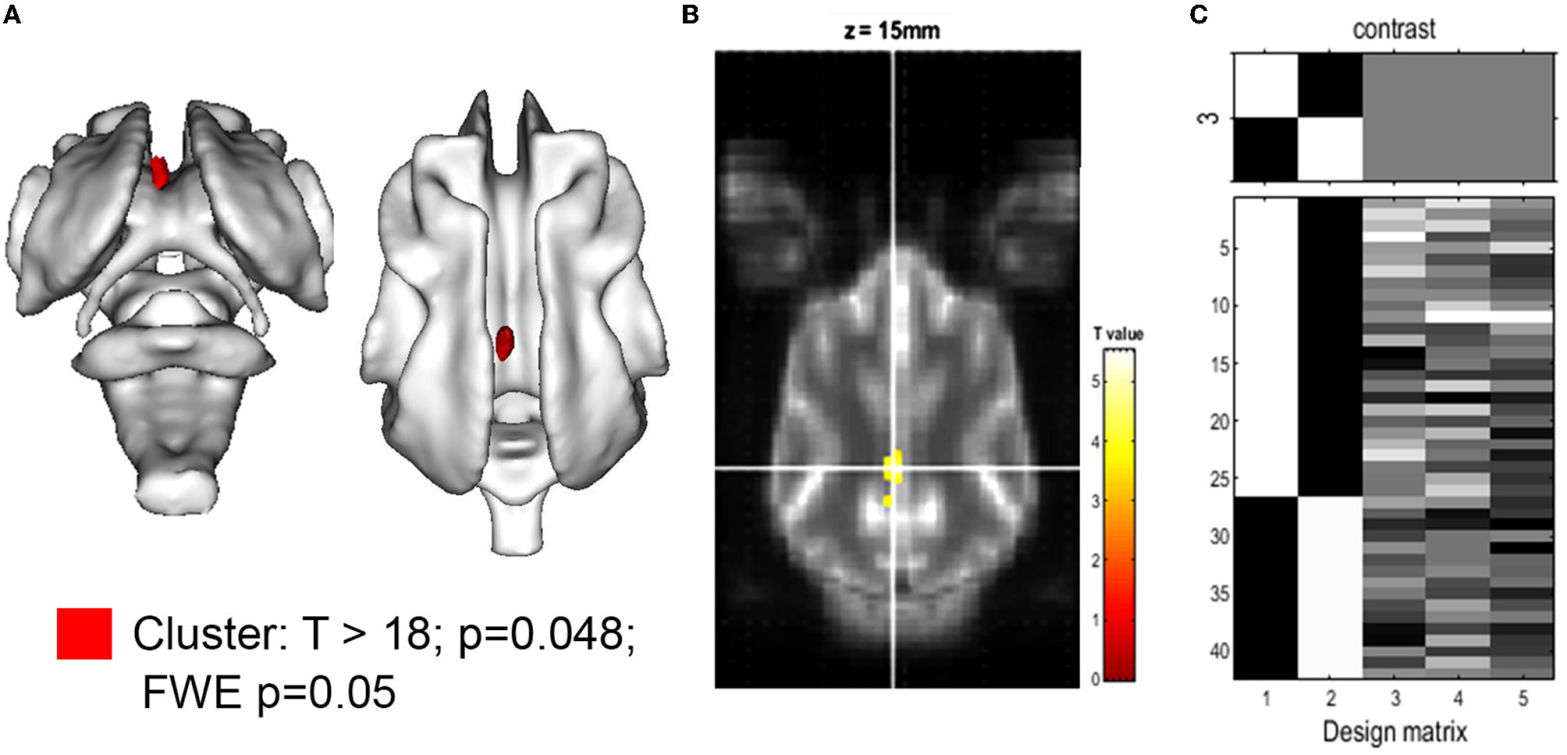
Voxel-based morphometry of the gray matter for the hypothesis: CKCS, SM, and pain < control. **(A)** 3D rendering of the cluster of significance (red, *p* = 0.048). The cluster was projected on the white matter of the canine brain atlas (Nitzsche et al., 2018) and is located in the cingulate gyrus. The *T*-value map with *T* >18 is projected onto the canine brain atlas as given by SPM. **(B)** A transverse atlas image with the cluster of significance overlayed at coordinates (*x, y, z*) [−1.5, −16, 14] as given by SPM12. The *T*-test between CKCS with SM and chronic pain controls was performed using family-wise error (few; *p* = 0.05) and brain volume, age, and body weight as covariance factors. **(C)** The design matrix **(right)** shows the number of individuals per group (assignment in the first 2 rows) as well as the value of the respective covariant for each individual (rows 3–5).

The comparison of relative brain structure volumes (in relation to individual brain volume) revealed no significant differences in any cerebral, cerebellar, or ventricle structure except for the volume of the CG, which was significantly smaller in CKCS with SM and pain. Details are presented in Table 2. The finding was supported by a statistical, voxel-wise comparison of GM masks (VBM) from control and CKCS with SM and pain (Figure 2). One cluster survived significantly at coordinates (*x, y*, and *z*) [−1.5, −16, 14] (*p* = 0.048). No significant voxels were detected when analyzing WM or the ventricular system.

**Figure 3:**
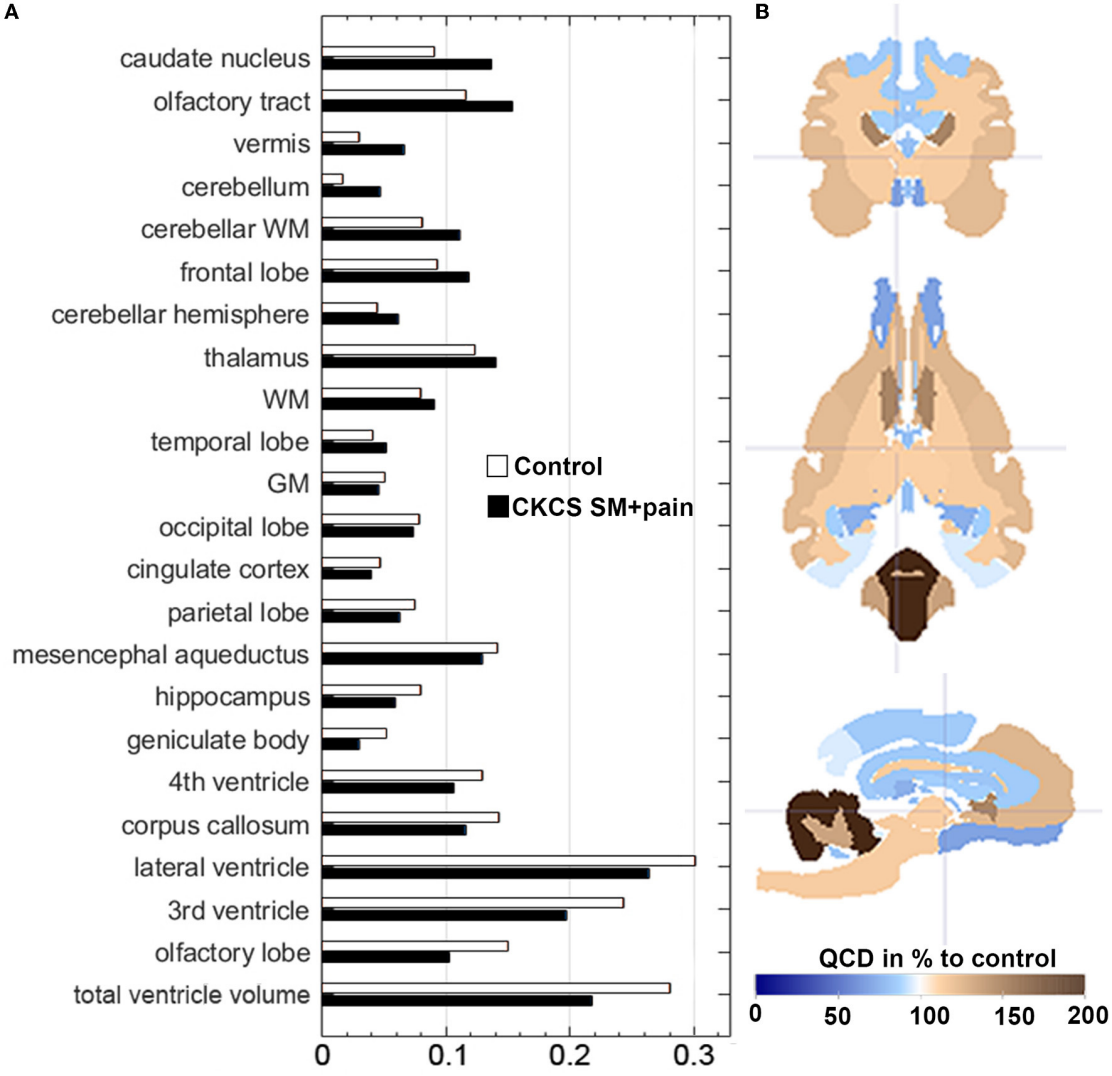
**(A)** Data distribution of relative brain structure volumes using the Quartile Coefficient of Dispersion (QCD). QCDs of relative brain structure volumes for CKCS with SM and pain vs. controls. The QCDs of relative cingulate cortex volumes were almost the same in both groups, while those of the cerebellum and the vermis were almost two-fold higher in CKCS with SM and pain than in controls. **(B)** The parametrized label map (based on atlas labels by Nitzsche et al., 2018) shows the percentual difference of CKCS with SM and pain QCDs compared to control QCDs. Crosshair at coordinates (*x, y, z*) [2, 2, 2].

The QCDs of relative GM volumes were almost the same in both groups (0.05 in control vs. 0.046 in CKCS with SM and pain), while the highest QCDs were generally found for total ventricle volumes (0.28 in controls vs. 0.22 in CKCS with SM and pain), and in particular the lateral (0.31) and 3rd ventricle volumes. Moreover, the QCDs of lateral and third ventricle volumes were higher in controls (0.31 and 0.24) as compared to CKCS with SM and pain (0.26 and 0.19). Interestingly, the QCDs of cerebellar volumes in CKCS with SM and pain (0.05) were larger than in controls (0.01). Specifically, there was a more than two-fold larger QCD for vermis volumes in CKCS with SM and pain (0.07) as compared to controls (0.03; Figure 3).

## Discussion

This is the first investigation documenting a potential relationship between SM with chronic pain and structural changes in cerebral pain network elements in CKCS. There was a small but significant volume decrease in the CG GM in CKCS with SM and chronic pain compared to controls.

The CG, in particular, the anterior part, is known to be a key area in central modulation processes involved in chronic pain sensation in humans (Woodworth et al., 2019). A decrease in CG GM was observed in humans with various chronic pain conditions (Apkarian et al., 2004; Rodriguez-Raecke et al., 2009). Several longitudinal studies using VBM have demonstrated the reversibility of GM atrophy after successful treatment of chronic pain (Rodriguez-Raecke et al., 2009; Obermann et al., 2013). This suggests that the decreased GM volume is not related to neuronal death. The changes in the size of dendritic spines, intercortical connections, and decrease in cell size were suggested to be the main factors contributing to the reduced GM volume (Rodriguez-Raecke et al., 2009). While atrophy has been detected in different parts of the human pain network, the CG and the prefrontal cortex were most consistently involved throughout studies (Rodriguez-Raecke et al., 2009). A study by Szabo et al. (2019) revealed that the CG is an element of a resting-state network in dogs, which also includes other areas such as the left premotor area, medial and bilateral caudal regions of the CG, and the splenial gyrus. However, the extent and type of potential morphological alterations in a pathological scenario in dogs remain unclear. In this pilot study, we show exclusive CG atrophy in the CKCS with SM and chronic pain by using 21 labeled brain regions. However, we could not exclude secondary degeneration of cerebral white matter by syringomyelia associated with pain. Thus, further prospective studies are required to identify network-dependent alterations in the pain network of syringomyelia (Czeibert et al., 2019; Johnson et al., 2020), including white matter tracts.

We could not detect differences in any cerebral volumes besides the CG or between any ventricle volumes when corrected for total brain volume. Furthermore, VBM did not detect any difference in ventricles between the two groups. Thus, we conclude that syringomyelia had only a minor effect on cerebral structures and, therefore, the exclusive appearance of CG changes in CKCS with SM and pain was indeed pain-related.

The accuracy of VBM for assessing small volume differences in neuroradiological datasets has been proven in humans, canines, and other species (Hellewell et al., 2019). For instance, VBM using a voxel-wise approach has been shown to be a powerful morphometric tool to investigate differences in brain volumes in dogs with epilepsy (Frank et al., 2018) and compulsive disorders (Ogata et al., 2013). However, the applicability of the technique when assessing canine datasets is limited due to the interbreed variability of canine brain structures. Thus, we considered known covariates such as skull morphology, brain size, body weight, and age (Schmidt et al., 2014). We used an approved image processing pipeline to enable nearly automated processing, avoiding observer interference by processing all data equally. Nevertheless, we cannot exclude having missed minor differences due to the relatively small group size in our study.

The accuracy of measuring brain substructure volumes by a series of cross-sectional MR images is limited by voxel size, slice thickness, and large interslice distances. Images retrospectively analyzed in this study were produced in routine clinical examinations, considering clinical needs such as the short duration of anesthesia. Thus, imaging settings were not optimized as could be expected in an experimental setting or a prospective trial. Of note, MR modality and image size are comparable to other studies (Abbott et al., 2012; Ogata et al., 2013; Frank et al., 2018; Czeibert et al., 2020). However, an interslice distance (spacing) of 2.2 mm and non-isovoxel data acquisition might limit the accuracy of our approach since the longitudinal extent of subcortical structures may be underestimated because of potential misalignments during the registration process of atlas labels. We have compensated for this drawback by reslicing with the 6th B-spline interpolation. Nevertheless, missing differences between groups, especially at the pole of a longitudinal brain structure, cannot be excluded. On the other hand, it was shown that interslice distances up to 5 mm do not markedly reduce accuracy or provide an obstacle to reliable comparisons in humans when using bicubic or bilinear interpolation as implemented in SPM (Ghoshal et al., 2020). Importantly, imaging protocols were identical for all dogs, which strongly limits potential inaccuracies in any assessment (Schmidt et al., 2015). Furthermore, prospective studies must address these issues by implementing a fast, sensitive, and high-resolution 3D MRI technique, including a shorter interslice distance.

The variability of brain structure volumes in our study was similar to our previous findings for single breeds (Nitzsche et al., 2018), except for the cerebellum. Interestingly, the relative cerebellar volume of CKCS with SM and pain varied two-fold more than in control dogs (Figure 3). This may explain the lack of statistical significance of cerebellar volume differences in our study in contrast to others (Shaw et al., 2012). Thus, the high variability may give a hint to underlying entities altering the cerebellum in CKCS with SM and pain. This needs to be clarified in further studies.

Significant differences in the corpus callosum and olfactory tract volumes were found when comparing absolute tissue volume (see Table 2). However, no differences could be detected when comparing volumes after adjusting for individual brain volumes. Indeed, publications on clinical cases report similar features in human CM patients (Erol, 2012; Tijssen et al., 2016). Unfortunately, a systematic analysis is still lacking to the best of our knowledge. It is reasonable not to exclude the possibility that a real difference in our study may have been covered by the relatively wide inter-individual data scatter and the small group sizes. If such differences exist, altered glymphatic circulation, inflammatory processes, and edema may indeed explain them. However, our data do not allow us to conclude that such differences indeed exist. Given the comparable clinical and morphometrical features of CM in CKCS and humans, we believe that further research in canine populations may significantly contribute to clarifying this question and potentially even to create a “model” to study the condition's impact on the brain morphometry in real-world veterinary patient populations. However, this would be left for future research.

There is a correlation between cortical GM atrophy and the duration of pain (Rodriguez-Raecke et al., 2009; Fuchs et al., 2014). In our study group, the average time of clinical symptoms being present was 8 months (range: 3.5–24 months), and chronic pain was diagnosed when it lasts or recurs for more than 3 months according to established standards (Merskey et al., 1994). Although it is not clear how much time is necessary for pain-associated brain atrophy to become macroscopically visible in dogs, it can be assumed that atrophy increases with the longer presence of chronic pain. The analysis of the brains of CKCS with long-lasting chronic pain (12 months or longer) could have potentially revealed volume differences in other cortical areas as well. However, only a minority of the dogs in our study group had chronic pain for more than 12 months (*n* = 3). One limitation of this study is the retrospective assessment of neuropathic pain, which is mainly based on the frequency of scratching attacks per day. Moreover, the intensity of perceived pain might have an influence on GM atrophy. Thus, a comparison of dogs with comparable chronic pain duration and intensity would be ideal. However, methods to objectively assess neuropathic pain intensity in CKCS with SM are lacking (Thoefner et al., 2019), and objectivity may further be hampered by the fact that assessment of pain is usually done by patient owners rather than veterinarians.

Similar to human patients, CKCS with SM can present comorbidities of chronic neuropathic pain such as lethargy, disturbed sleep, decreased willingness to exercise, and increased fear-related behavior (Sanchis-Mora et al., 2016; Rusbridge et al., 2019). Such comorbidities are another potential explanation for the differences observed between dogs in the study group and control dogs. Thus, we cannot exclude that CG atrophy may have been caused by comorbidities or a combination of pain and comorbidities. However, only two of the owners reported typical comorbidities in their dogs (lethargy and nighttime wandering), somewhat mitigating this concern.

At the present stage, the impact of our results may not go beyond the first tentative evidence for a “signature of chronic pain” in canine brains. Notwithstanding the potential limitations, our data warrants a more detailed investigation of pain-related brain atrophy in dogs as well as potential factors being causative or contributing to this process in larger, potentially multi-center clinical studies. If CG atrophy is consistently found in these studies, it may serve as an objective parameter for the diagnosis of neuropathic pain, as an indication for therapeutic intervention, or as an objective readout parameter for therapeutic efficiency in canine pain syndromes.

## Conclusion and clinical relevance

CG GM volumes are decreased in CKCS with SM and chronic pain, as shown by comparing relative volumes and VBM. If confirmed in future studies, CG-GM atrophy may serve as an objective parameter for the diagnosis of neuropathic pain, as an indication for therapeutic intervention, or as an objective readout parameter for therapeutic efficiency in canine pain syndromes.

## Data availability statement

The original contributions presented in the study are included in the article/Supplementary material, further inquiries can be directed to the corresponding author.

## Ethics statement

Ethical review and approval was not required for the animal study because, Retrospective study: approval from the Ethics Committee of the Justus Liebig University and the responsible regional governmental authority (Regierungspräsidium) of the federal state of Hesse was not sought because the authority waives ethical review of retrospective studies based on clinical imaging conducted for diagnostic purposes. Written informed consent was obtained from the owners for the participation of their animals in this study.

## Author contributions

BN drafted the manuscript, generated, wrote and discussed the results, coordinate the manuscript writing, contributed to all paragraphs, and created figures and tables. SS drafted the manuscript, collect data and worked on data, discussed the results, and contributed to method section. JB drafted the mansucript, wrote the manuscript, discussed results, designed it's structure, and contributed to all paragraphes. MS drafted the manuscript idea, designed its structure, wrote manuscript, and contributed the majority of the paragraphes. All authors contributed to the article and approved the submitted version.
