# Supplementary material for: Reduced cingulate gyrus volume in Cavalier King Charles Spaniels with syringomyelia and neuropathic pain revealed by voxel-based morphometry: a pilot study
Source: Front Neuroanat. 2023 Jul 17;17:1175953. doi: 10.3389/fnana.2023.1175953 (PMC10389659; doi:10.3389/fnana.2023.1175953)
Supplement: Supplementary file 1 [file Table_1.DOCX]

Supplements

Supplementary table 1

| Dog NO | **Age in months** | **Sex** | **Body weight** | **Diagnosis** | **duration of signs (months)** | **scratching per day** |
| --- | --- | --- | --- | --- | --- | --- |
| **1** | 60 | f | 10.5 | SM | 10 | 3 |
| **2** | 156 | f | 8.1 | SM | 24 | 6 |
| **3** | 72 | f | 11 | SM | 6 | 6 |
| **4** | 72 | m | 9.5 | SM | 6 | 5 |
| **5** | 36 | m | 8.1 | SM | 3.5 | 0.7 |
| **6** | 48 | f | 7.8 | SM | 24 | 12 |
| **7** | 84 | f | 7.5 | SM | 6 | 6 |
| **8** | 36 | m | 8.5 | SM | 3 | 4 |
| **9** | 84 | m | 11 | SM | 12 | 4 |
| **10** | 120 | m | 10.2 | SM | 6 | 3 |
| **11** | 60 | m | 8 | SM | 3 | 0.7 |
| **12** | 36 | m | 8.6 | SM | 6 | 3 |
| **13** | 60 | m | 8 | SM | 6 | 5 |
| **14** | 36 | m | 11 | SM | 6 | 6 |
| **15** | 48 | m | 9.1 | SM | 3.5 | 5 |
| **16** | 24 | m | 10.5 | SM | 6 | 6 |
| **17** | 48 | m | 8.7 | SM | 4 | 6 |
| **18** | 36 | m | 9.3 | SM | 12 | 5 |
| **19** | 60 | f | 8.2 | SM | 6 | 5 |
| **20** | 12 | f | 7.5 | SM | 8 | 6 |
| **21** | 48 | f | 9.2 | SM | 24 | 7 |
| **22** | 36 | m | 7.8 | SM | 7 | 6 |
| **23** | 48 | m | 9.3 | SM | 6 | 3 |
| **24** | 48 | m | 10 | SM | 5 | 6 |
| **25** | 72 | f | 9.2 | SM | 6 | 5 |
| **1** | 36 | f | 8.8 | none | N/A | N/A |
| **2** | 24 | f | 9.3 | none | N/A | N/A |
| **3** | 36 | f | 10 | none | N/A | N/A |
| **4** | 60 | f | 8.9 | none | N/A | N/A |
| **5** | 36 | m | 7.5 | none | N/A | N/A |
| **6** | 36 | m | 8 | none | N/A | N/A |
| **7** | 48 | m | 9.5 | none | N/A | N/A |
| **8** | 24 | m | 6.9 | none | N/A | N/A |
| **9** | 36 | m | 6.6 | none | N/A | N/A |
| **10** | 60 | m | 10.1 | none | N/A | N/A |
| **11** | 36 | m | 10.9 | none | N/A | N/A |
| **12** | 60 | m | 8.5 | none | N/A | N/A |
| **13** | 36 | f | 10.4 | none | N/A | N/A |
| **14** | 72 | f | 11.5 | none | N/A | N/A |
| **15** | 60 | m | 11.2 | none | N/A | N/A |
| **16** | 60 | f | 9.8 | none | N/A | N/A |

Supplementary table 1: Age in months is given at time of diagnosis with MRI; f/m – female/male; SM – syringomyelia; N/A – not applicable
